# Genomic Insights into the Pathogenicity of Hypervirulent *Aeromonas hydrophila* Strain D4 Isolated from Diseased Blunt Snout Bream with the Epidemic Sequence Type 251 Clones

**DOI:** 10.3390/pathogens14060570

**Published:** 2025-06-06

**Authors:** Li Xu, Xingyu Kang, Zhicheng Wang, Zuyuan Xiao, Yi Luo

**Affiliations:** 1 College of Fisheries, Key Lab of Freshwater Animal Breeding, Ministry of Agriculture and Rural Affairs/Key Lab of Agricultural Animal Genetics, Breeding and Reproduction of Ministry of Education/Engineering Research Center of Green Development for Conventional Aquatic Biological Industry in the Yangtze River Economic Belt, Ministry of Education, Huazhong Agricultural University, Wuhan 430070, China; li_xu@webmail.hzau.edu.cn (L.X.); kangxingyu_1996@yeah.net (X.K.); wzc01997@gmail.com (Z.W.); 2022308120088@webmail.hzau.edu.cn (Z.X.); 2 State Key Laboratory of Agricultural Microbiology, Huazhong Agricultural University, Wuhan 430070, China

**Keywords:** *Aeromonas hydrophila*, ST251, comparative genomics, virulence factors, pathogenic mechanism

## Abstract

*Aeromonas hydrophila* ST251 is a crucial pathogen responsible for the outbreaks of Motile *Aeromonas* Septicemia (MAS) in global aquaculture. To elucidate the genetic basis underlying its hypervirulence, we investigated strain D4, an ST251 isolate recovered from diseased blunt snout bream. Phenotypic assays revealed that, compared to the environmental strain ATCC 7966^T^, D4 exhibited enhanced motility, hemolytic activity, and protease production. Average nucleotide identity (ANI) analysis demonstrated that D4 clustered within a distinct ST251 clade, with ANI values ≥ 99.74%. Comparative genomic analysis of D4, nine additional ST251 strains, and ATCC 7966^T^ identified multiple unique genomic islands in ST251 strains, including pathways for *myo*-inositol and L-fucose utilization and a pseudaminic acid biosynthesis gene cluster. These genetic elements are associated with nutrient acquisition and flagellar assembly, potentially enhancing colonization and environmental adaptability. In addition, distinct plasmids and prophages in ST251 strains may contribute to host adaptation and virulence by regulating stress responses and virulence-associated genes. These findings offer new insights into the molecular mechanisms driving the pathogenicity and adaptability of hypervirulent *A. hydrophila* ST251 strains.

## 1. Introduction

Aquaculture is one of the fastest-growing food production sectors worldwide, playing a vital role in meeting the increasing global demand for animal protein. The aquaculture industry contributes significantly to economic development and food security. With the expansion of farming scale and diversification of cultured species, aquaculture production continues to increase; however, it also faces challenges such as disease control and environmental pollution [1].

*Aeromonas hydrophila* is a Gram-negative opportunistic pathogen ubiquitously distributed in diverse aquatic environments. It is capable of infecting a broad range of hosts, including fish, amphibians, reptiles, and mammals [2,3]. In recent years, outbreaks of Motile *Aeromonas* Septicemia (MAS) in fish, caused by sequence type (ST) 251 *A. hydrophila*, have emerged as a significant challenge for the aquaculture industries in China and the United States [4,5]. These ST251 strains exhibit not only strong virulence but also notable epizootic potential. Comparative genomic analyses have revealed that epidemic isolates from diseased fish in Asia and the United States share highly similar genomes, suggesting a common origin and global dissemination through waterborne transmission or the international trade of aquatic animals [6].

The pathogenic mechanism of *A. hydrophila* is complex, involving a variety of virulence factors. In previous research, a number of virulence factors, including secretion systems, motility and adhesins, toxins, enzymes, quorum systems, iron acquisition, and antibiotic resistance, have been identified [7]. However, the detailed pathogenesis of *A. hydrophila* is still unclear.

Studying the whole genome of *A. hydrophila* can provide valuable insights into its pathogenic mechanism. We hypothesized that unique genetic features in ST251 strains contribute to their enhanced virulence and environmental adaptability. In our previous work, we sequenced the genome of hypervirulent ST251 strain D4, which was isolated from diseased blunt snout bream (*Megalobrama amblycephala*) [8]. Building on this, the present study aims to (1) characterize the virulence phenotypes of strain D4 in comparison with the environmental isolate ATCC 7966^T^ and (2) perform a comparative genomic analysis of D4 with other ST251 strains and environmental strains. Our findings reveal distinct genomic features, including specific metabolic pathways and virulence-associated gene clusters, plasmids, and prophages, that may underpin the enhanced pathogenicity and host adaptation of ST251 strains.

## 2. Materials and Methods

### 2.1. Strains and Genomes

In this study, the virulence phenotype of strain D4 was characterized. To investigate the genetic determinants of hypervirulence in strain D4 and other *A. hydrophila* ST251 isolates, comparative genomic analyses were performed on nine epidemic strains from China and the United States, along with the strain ATCC 7966^T^ (ST1).

*A. hydrophila* strain D4 was isolated from the liver of one of three moribund blunt snout breams (*Megalobrama amblycephala*: a body length of 15–18 cm and a weight of 90–110 g), collected during a MAS outbreak at a fish farm in Wuhan, China. The genomes of *A. hydrophila* strain D4 was sequenced and submitted to GenBank with accession numbers CP013965-CP013969 [8].

Ten *A. hydrophila* strains, namely D4, JBN2301, ZYAH72, NJ-35, J-1, GYK1, ML09-119, AL09-71, pc104A and ATCC 7966^T^, were used in this study to perform the comparative genomic analyses (Table 1). The 19 genomes (*A. hydrophila*: YL17, 23-C-23, WCX23, AHNIH1, AL06-06, AH10, MX16A, WCHAH045096, GSH82, KN-Mc-1R2, ZYAH75, and 4AK4; *Aeromonas dhakensis*: KN-Mc-6U21; *Aeromonas sobria*: CECT 4245; *Aeromonas veronii*: B565; *Aeromonas salmonicida* subsp. *Salmonicida*: A449; *Aeromonas caviae*: 429865; *Aeromonas media*: WS; and *Aeromonas rivipollensis*: KN-Mc-11N1) used to perform the average nucleotide identity (ANI) analysis were deposited in GenBank with the following accession numbers: NZ_CP007518, NZ_CP038465, NZ_CP038463, NZ_CP016380, NZ_CP010947, NZ_CP011100, NZ_CP018201, NZ_CP028568.2, NZ_AP019193, NZ_CP027804, NZ_CP016990, NZ_CP006579, NZ_CP023141, NZ_CDBW01000006, NC_015424, NC_009348, NZ_LIIX01000004, NZ_CP007567, and NZ_CP027856, respectively.

### 2.2. Phenotypic Identification of Strain D4

#### 2.2.1. Swimming and Swarming Motility Assays

Motility assays were conducted in swimming and swarming media. The 0.3% agar LB swim medium consisted of 10 g of tryptone, 5 g of yeast extract, 10 g of NaCl, and 3 g of agar in 1 L of deionized water. The 0.5% agar LB swarm medium contained 10 g of tryptone, 5 g of yeast extract, 10 g of NaCl, and 5 g of agar in 1 L of deionized water. A 5 µL aliquot of D4 and ATCC 7966^T^ cultures at an OD_600_ of 0.3 was placed in the center of each plate. The plates were covered and allowed to stand for 5 min. Then, the plates were incubated at 28 °C for 24 h. The diameters of bacterial swimming and swarming zones were measured.

#### 2.2.2. Hemolytic and Protease Activity Assays

Hemolytic activity: Cultures of *A. hydrophila* D4 and ATCC 7966^T^, which were grown in the LB medium at 28 °C with shaking at 180 rpm for 18 h, were treated with trypsin (final concentration: 0.05%) at 28 °C for 1 h. The number of hemolytic units per milliliter of cell filtrate per 1 × 10^8^ colony-forming units (CFU) was determined and reported. The culture supernatants were mixed with rabbit red blood cells (2%) in a 96-well plate at a volume ratio of 1:3. The plate was incubated at 37 °C for 1 h and subsequently at 4 °C overnight. Hemolytic activity was evaluated by measuring the absorbance of the mixture supernatants at 540 nm.

Protease activity: An aliquot (200 μL) of overnight culture filtrates from *A. hydrophila* D4 and ATCC 7966^T^ was added to disposable 5 mL snap-cap tubes. Each tube contained 800 μL of DPBS and 5 mg of the Hide azure powder substrate. The tubes were incubated in a shaker incubator at 37 °C for 3 h. As the proteinase in the culture filtrates catalyzed the substrate, a blue color was released. The intensity of the blue color was quantified at an optical density of 595 nm (OD_595_). The proteinase activity was calculated as the amount per milliliter of culture filtrate per 1 × 10^8^ CFU. The substrate incubated with the LB medium alone served as a negative control.

#### 2.2.3. Biofilm Formation Assay

Ten microliters of overnight-grown bacterial cultures (1 × 10^8^ CFU/mL) of *A. hydrophila* D4 and ATCC 7966^T^ was transferred into a well containing 190 μL of the LB medium in a 96-well plate and incubated under static conditions at 28 °C for 48 h. The medium was carefully aspirated, and the well was then washed three times with deionized water to remove any non-adherent cells. The biofilm was stained with a 1% crystal violet solution for 15 min. Subsequently, the excess stain was thoroughly rinsed off with deionized water. The dye bound to the biofilm structure was extracted with pure ethanol, and the absorbance of the resulting solution at 570 nm was measured.

Iron utilization: Briefly, siderophores produced by the bacterial strains were analyzed based on the chrome azurol-S (CAS) analytical method. To prepare the inoculum, bacterial strains were first grown in the sterilized LB medium at 28 °C for 18 h on a rotary shaker set at 180 rpm. Then, the cells were harvested by centrifugation at 5000 rpm for 10 min. The supernatant was mixed with the CAS Assay Solution. A reference sample was prepared by mixing the CAS Assay Solution with the non-inoculated LB medium. Absorbances at 630 nm were determined, and values were compared with those of the reference. The OD value at 570 nm in the experimental group was recorded as ODe and the negative control group as ODc. We adopted the well-established evaluation criteria for biofilm formation ability from the work by Davey and O’toole [9]: non-adhesive when ODc ≤ Ode, weakly adhesive when ODc < ODe ≤ 2ODc, moderately adhesive when 2ODc < ODe ≤ 4ODc, and strongly adhesive when ODe > 4ODc.

#### 2.2.4. Lethal Dose 50 Assay

Single colonies of each bacterial strain were inoculated into LB broth. The resulting overnight cultures were then diluted to a ratio of 1:100 in fresh LB broth. The inoculated cultures were incubated at 28 °C with shaking at 180 rpm until they reached the logarithmic growth phase, which was determined by measuring the optical density at OD_600_ until they reached 0.6. The harvested cells were washed twice with phosphate-buffered saline (PBS) and then resuspended in PBS to achieve appropriate concentrations.

For each isolate, five groups, each consisting of ten fish, fish were intraperitoneally injected with 0.02 mL of serially ten-fold diluted bacterial suspensions. The suspensions had concentrations ranging from 1 × 10^4^ to 1 × 10^8^ CFU. Another group of ten zebrafish (serving as the control group) was intraperitoneally injected with 0.02 mL of sterile PBS only.

The zebrafish were placed in separate tanks, with one tank dedicated to each bacterial infection group. The tanks were aerated and had a non-circulating water system, and the water temperature was maintained at 28 °C. The zebrafish were observed for one week post-infection. Water was occasionally added to account for evaporation, and any dead fish were removed from the tanks during the course of the experiments. For each determination, three independent experiments were conducted. Survival data were analyzed by the method of Reed and Muench for the calculation of LD_50_ values [10].

### 2.3. Statistical Analyses

All experiments were replicated at least three times. In all experiments, the data are presented as the mean ± standard deviation. Results were analyzed using one-way analysis of variance (ANOVA) followed by a post hoc Tukey’s test with the Statistical Package for the Social Sciences (SPSS) 26.0 software.

### 2.4. Comparative Genomics Analysis

#### 2.4.1. ANI Analysis and General Features of Ten *A. hydrophila* Genomes

To further determine the classification of strain D4, the average nucleotide homology (ANI) among 29 *Aeromonas* spp. genomes was analyzed using the built-in ANIb program JSpecies (version 1.2.1).

#### 2.4.2. Genomic Collinearity and Rearrangement Analysis

To explore the genomic structure and collinearity of 10 strains of *A. hydrophila* ST251, the linear structure of 10 genomes was compared by progressive MAUVE (version 2.3.1), with the genome of strain D4 serving as the reference genome.

#### 2.4.3. Functional Annotation and Pathway Analysis

To classify the functions encoded in the 10 *A. hydrophila* genomes, BLASTp was used to align the amino acid sequences against the database of Clusters of Orthologous Groups (http://www.ncbi.nlm.nih.gov/COG/ (accessed on 20/05/2024)). The *A. hydrophila* genomes were submitted to the KEGG pathway database (http://www.genome.jp/kegg/pathway.html (accessed on 10/06/2024)) for annotation.

### 2.5. Virulence-Related Factors Analysis

#### 2.5.1. Virulence Gene Conservation and Absence Analysis

To comprehensively identify and analyze the virulence genes present in 10 *A. hydrophila* strains, BLASTp was applied. The amino acid sequences of the proteins encoded by the genomes of these 10 strains were aligned against the Virulence Factor Database (VFDB) to annotate the virulence genes.

#### 2.5.2. Secondary Metabolite Gene Cluster Analysis

To explore the potential production of secondary metabolites by 10 *A. hydrophila* strains, the prediction of their secondary metabolite gene clusters was performed using the antiSMASH 5.0 online tool with default parameters (https://antismash.secondarymetabolites.org/ (accessed on 01/07/2024)) [11].

#### 2.5.3. Prophage Content and Functional Analysis

To investigate the prophage elements harbored within 10 *A. hydrophila* strains, the prediction of prophages in these strains was conducted using the PHASTER online tool with default parameters (http://phaster.ca/ (accessed on 28/01/2025)).

#### 2.5.4. Genomic Islands Analysis

To identify potential genomic islands that may contribute to the unique characteristics of 10 *A. hydrophila* strains, the prediction of genomic islands in these 10 strains was performed using IslandViewer 4 with default parameters (http://pathogenomics.sfu.ca/islandviewer (accessed on 12/02/2025)).

#### 2.5.5. Plasmid Content and Functional Analysis

The collinearity comparison between pAhD4-1 (accession number: CP013966.1) and pHX3 (accession number: CP040718.1) was drawn using Easyfig (version 2.2.5) by inputting their GenBank files, performing BLAST alignment, and configuring parameters to analyze homologous regions and genetic distribution differences between the two plasmids.

## 3. Results

### 3.1. Phenotypic Characterization of Strain D4

#### 3.1.1. Swimming and Swarming Motility

**Figure 1:**
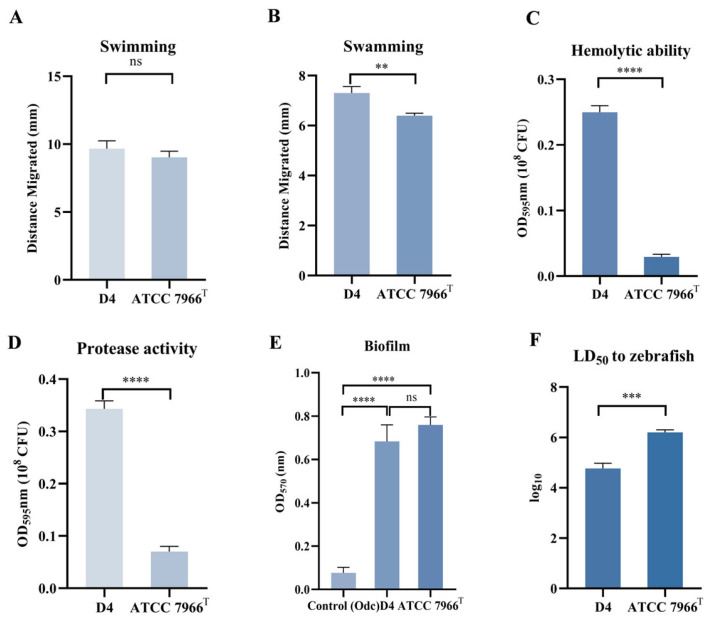
Phenotypic characterization of strain D4. (**A**) Swimming motility of *A. hydrophila* strains D4 and ATCC 7966^T^. (**B**) Swarming motility of *A. hydrophila* strains D4 and ATCC 7966^T^. (**C**) Hemolytic activity in the culture supernatants of *A. hydrophila* strains D4 and ATCC 7966^T^. (**D**) Protease activity in the culture supernatants of *A. hydrophila* strains D4 and ATCC 7966^T^. (**E**) Biofilm formation of *A. hydrophila* strains D4 and ATCC 7966^T^. (**F**) Virulence of *A. hydrophila* assessed in zebrafish. Three independent experiments were conducted, and the data presented are the arithmetic means ± standard deviations. ** indicates a significant difference (*p* < 0.01); *** indicates a significant difference (*p* < 0.001); **** indicates a significant difference (*p* < 0.0001).

Flagella are complex surface organelles that enable bacteria to move towards favorable environments and contribute to the virulence of pathogenic bacteria via adhesion and biofilm formation on host surfaces. However, only a limited number of bacteria have dual flagella systems: a polar flagellum for swimming and lateral flagella for swarming [12,13]. To detect the difference in motility phenotypes between the epidemic ST251 strain D4 and the environmental strain ATCC 7966^T^, the 0.3% agar LB medium was used to assess the swimming ability of bacteria, and the 0.5% agar LB medium was employed to evaluate the swarming movement of bacteria. From the results of the swimming motility assay (Figure 1A), there was no significant difference in the migrated distance of colonies between D4 and ATCC 7966^T^ (*p* > 0.05). However, in the swarming motility assay, the migrated distance of colonies of ATCC 7966^T^ was significantly shorter than that of D4 (*p* < 0.01) (Figure 1B).

#### 3.1.2. Hemolytic and Protease Activity

*A. hydrophila* can produce a variety of extracellular products, such as toxins (mainly including aerolysin, cytotoxic enterotoxin, and hemolysin) and extracellular proteases, most of which are associated with bacterial pathogenicity [14,15,16]. The hemolytic and protease activities of the epidemic strain D4 and the environmental strain ATCC 7966^T^ were compared using the methods described above. As shown in Figure 1C,D, the hemolytic and protease activities of the extracellular products of strain D4 were significantly higher than those of ATCC 7966^T^ (*p* < 0.0001).

#### 3.1.3. Biofilm

Biofilm formation ability was evaluated using the 96-well plate method. The biofilm-forming abilities of the epidemic strain D4 and environmental strain ATCC 7966^T^ are shown in Figure 1E. No significant difference was observed in biofilm formation between the two strains, but both displayed significantly higher biofilm formation than the control (*p* < 0.0001). Specifically, the ODe values of both strains were greater than 4ODc, indicating that both the epidemic strain D4 and the environmental strain ATCC 7966^T^ have strong biofilm-forming capabilities.

#### 3.1.4. Virulence Assessment via LD_50_ Determination

To evaluate the virulence of *A. hydrophila* strains D4 and ATCC 7966^T^, the median lethal dose (LD_50_) in zebrafish (*Danio rerio*) was determined. As shown in Figure 1F, the LD_50_ values for D4 and ATCC 7966^T^ were 4.59 × 10^4^ CFU/fish and 1.65 × 10^6^ CFU/fish, respectively. According to the virulence classification criteria established by Rodríguez et al. [17], strain D4 was categorized as virulent (LD_50_ < 1.0 × 10^6^ CFU/fish), whereas the environmental isolate ATCC 7966^T^ was classified as avirulent (LD_50_ > 1.0 × 10^6^ CFU/fish).

### 3.2. Comparative Genomics Analysis

#### 3.2.1. ANI Analysis and General Features of Ten *A. hydrophila* Genomes

**Figure 2:**
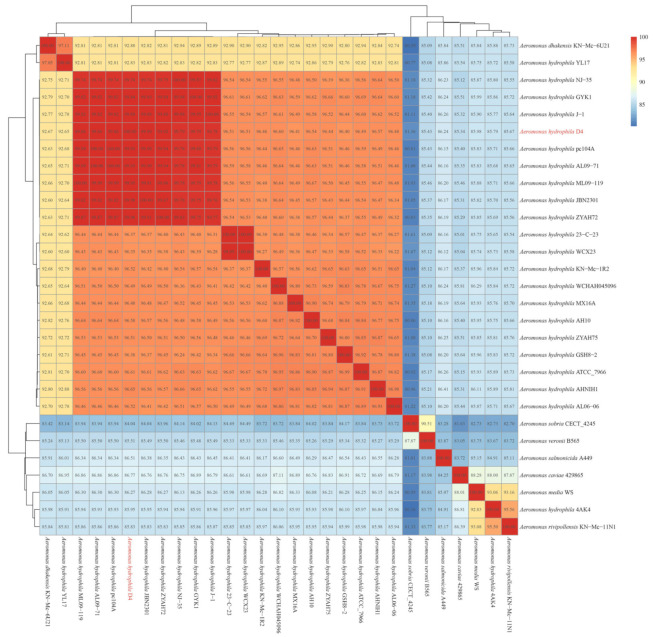
Average nucleotide identity of 29 strains of *Aeromonas*.

To further determine the classification of strain D4, ANI was calculated among 29 *Aeromonas* spp. genomes deposited in GenBank (Figure 2). As previously established, species-level classification requires ANI ≥ 95% [18]. Strain D4 clustered closely with *A. hydrophila* reference genomes, yielding ANI values >95%, confirming its classification as *A. hydrophila*. Conversely, strains YL17 and 4AK4 showed ANI values ≤ 92.88% and ≤ 86.04% with *A. hydrophila* genomes, respectively, suggesting potential misclassification. Subsequent analysis revealed that YL17 shared 97.1% ANI with *A. dhakensis* KN-Mc-6U21 and 4AK4 shared 95.5% ANI with *A. rivipollensis* KN-Mc-11N1, supporting their reclassification into these species. Notably, *A. hydrophila* strains D4, ZYAH72, NJ-35, J-1, GYK1, JBN2301, ML09-119, AL09-7, and pc104A displayed higher ANI values compared to other *A. hydrophila* genomes, clustering together into a distinct clade with ANI ≥ 99.74% and all belonging to ST251.

**Figure 3:**
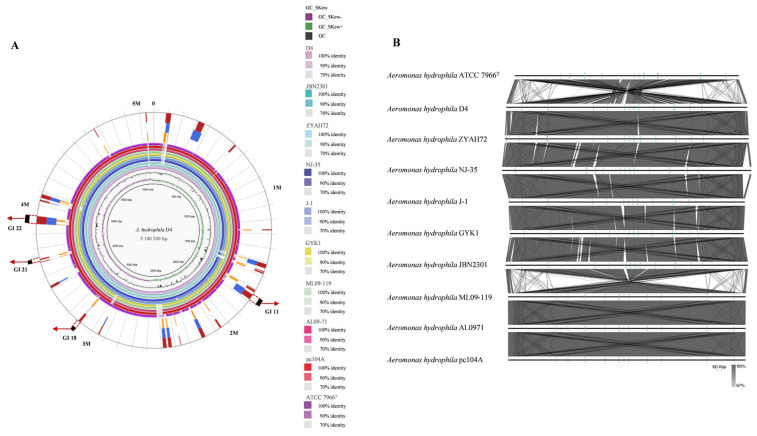
Whole-genome analysis of *A. hydrophila*. (**A**) Circular representation of ten *A. hydrophila* genome. The arrow indicates the genomic island. Red square: integrated prediction methods; blue square: IslandPath-DIMOB prediction method; orange square: SIGI-HMM prediction method. (**B**) Collinear analysis result of nine *A. hydrophila* strains and the environmental isolate ATCC 7966^T^.

A whole-genome overview is shown in Table 1. Among the ten strains studied, D4 (this study), ZYAH72 [19], NJ-35 [20], J-1 [21], GYK1 [22], and JBN2301 [23] were the epidemic strains collected in China; ML09-119 [24], AL09-71 [25], and pc104A [26] were the epidemic strains isolated in the United States; and ATCC 7966^T^ [27] was an environmental strain. There were four and three plasmids in the D4 and JBN2301 genomes, respectively, and no plasmid was found in other genomes. All ten strains harbored a single circular chromosome, with sizes ranging from 4.74 Mb (ATCC 7966^T^) to 5.28 Mb (NJ-35). Notably, the genome of the environmental isolate ATCC 7966^T^ was significantly smaller than those of the epidemic strains. The genome G + C content was conserved (60.50–61.51%), but coding sequences (CDSs) varied from 4151 (ATCC 7966^T^) to 4569 (D4). All strains encoded 31 rRNAs, while tRNA, ncRNA, and pseudogene counts differed significantly. As shown in Figure 3A, comparative analysis revealed extensive CDS deletions in ATCC 7966^T^ relative to ST251 strains, suggesting reduced functional complexity in the environmental isolate.

#### 3.2.2. Genomic Collinearity and Rearrangement Analysis

To investigate the genomic structure and collinearity among ten *A. hydrophila* strains, progressive MAUVE alignment was performed using strain D4 as a reference genome (Figure 3B). Notably, no genome rearrangements were detected among the six Chinese epidemic strains (D4, ZYAH72, NJ-35, J-1, GYK1, and JBN2301) or within the three American epidemic strains (ML09-119, AL09-71, and pc104A). However, significant rearrangements were observed between the Chinese and American epidemic lineages, as well as between the environmental isolate ATCC 7966^T^ and all epidemic strains.

The majority of genomic rearrangements were characterized by large-scale inversions of chromosomal segments. Previous studies have highlighted that such inversions play critical roles in adaptive evolution and ecological divergence [28]. The observed inversions between the Chinese and American epidemic clades may reflect geographically specific environmental adaptations, potentially contributing to their distinct epidemiological profiles.

#### 3.2.3. Functional Annotation and Pathway Analysis

**Figure 4:**
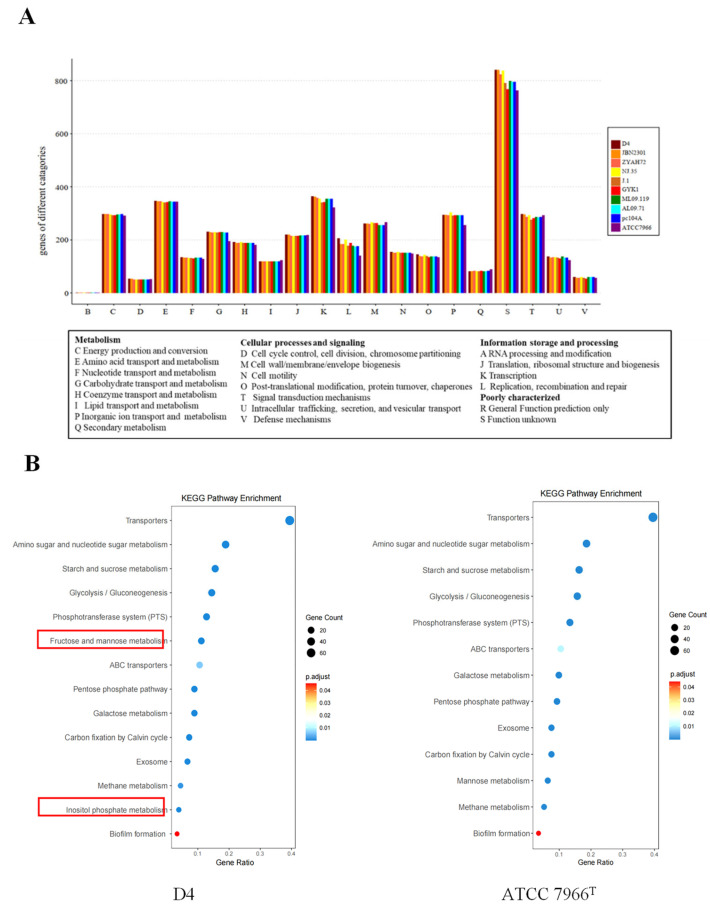
Functional annotation and pathway analysis. (**A**) COG functional categories among ten *A. hydrophila* genomes. (**B**) KEGG pathway enrichment of COG category G genes in D4 and ATCC 7966^T^. The red box indicates that this pathway is present in strain D4 but absent in ATCC 7966^T^.

To identify functional determinants underlying virulence diversity in epidemic strains, ten *A. hydrophila* genomes were annotated against the Clusters of Orthologous Groups (COG) database. As shown in Figure 4A, the environmental isolate ATCC 7966^T^ harbored significantly fewer genes in COG categories G (carbohydrate transport and metabolism), K (transcription), L (replication/recombination/repair), P (inorganic ion transport), and S (function unknown) compared to the nine epidemic ST251 strains. This observation suggests that genes in these COG categories may contribute to the enhanced virulence of epidemic strains.

KEGG pathway analysis was performed on genes categorized under COG G, K, L, and P. Notably, genes involved in the *myo*-inositol and L-fucose metabolic pathways (COG G) were uniquely present in ST251 strains, as shown in Figure 4B (red box). The enrichment of *myo*-inositol and L-fucose metabolism in ST251 strains may enhance host colonization by providing energy sources during infection [29].

Similarly, ST251-specific genes in COG L encoded transposases and helicases, including *rapA*, which encodes an ATP-dependent helicase. Previous studies have linked *rapA* homologs in *Escherichia coli* to biofilm formation, multidrug resistance, and stress adaptation [30]. The exclusive presence of *rapA* in ST251 strains suggests its potential role as a virulence determinant.

### 3.3. Virulence-Related Factors Analysis

#### 3.3.1. Virulence Gene Conservation and Absence Analysis

**Figure 5:**
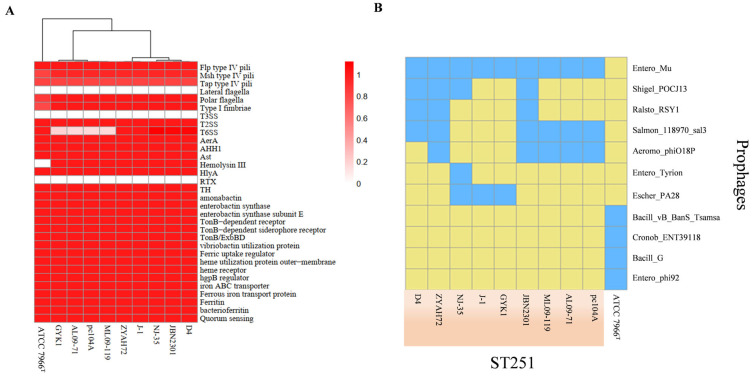
Virulence-related factors analysis. (**A**) Distribution of virulence factors among the *A. hydrophila* strains. (**B**) Type of prophages present across ten *A. hydrophila* strains. The blue color represents the presence of the prophage, and the yellow color represents the absence of the prophage, Orange represents the strains of *A. hydrophila* ST251.

Previous studies have demonstrated that virulence factors are conserved among closely related bacterial species [31], and our study also revealed that nearly all the virulence genes from the VFDB were conserved across the ten *A. hydrophila* strains, including strain D4. However, the type III secretion system (T3SS), the repeat in toxin (RTX) system, and the lateral flagella system were absent not only in the environmental strain ATCC 7966^T^ but also in the nine epidemic ST251 strains (Figure 5A). Additionally, the type VI secretion system (T6SS) was not present in three American strains (ML09-119, AL09-71, and pc104A) and one Chinese strain, GYK1. Intriguingly, although the T6SS was lacking in these four strains, the effector proteins (VgrG and Hcp) were detected. The above analyses suggest that the T3SS, the T6SS, the RTX system, and the lateral flagella system may not be essential virulence factors for the ST251 strain of *A. hydrophila*.

#### 3.3.2. Secondary Metabolite Gene Cluster Analysis

Secondary metabolites are synthesized during specific growth phases that are dependent on primary metabolic pathways and typically play non-essential roles in microbial survival and reproduction [32]. As shown in Table 2, no differences were observed in the types of secondary metabolite gene clusters between epidemic ST251 strains and the environmental isolate ATCC 7966^T^. All strains harbored four conserved clusters: arylpolyene, bacteriocin, non-ribosomal peptide synthetase (NRPS), and hserlactone. Notably, ten *A. hydrophila* strains shared Clusters 1–4, while Cluster 5 was uniquely present in strain NJ-35.

Cluster 3 displayed 100% similarity to the amonabactin P750 biosynthesis cluster in *A. hydrophila* ATCC 7966^T^, which is associated with siderophore production [27]. Cluster 1, encoding an arylpolyene synthase, shared 61% homology with reported arylpolyene clusters in *Xenorhabdus doucetiae*. Arylpolyenes are widespread Gram-negative bacterial secondary metabolites with roles in oxidative stress resistance [33]. The conservation of the arylpolyene and siderophore clusters across all strains suggests their roles in fundamental survival processes, potentially contributing to ecological fitness rather than strain-specific virulence.

Clusters 2 and 4 lacked significant homology to previously characterized clusters, warranting further functional investigation.

#### 3.3.3. Prophage Content and Functional Analysis

Prophages can enhance the pathogenicity of host bacteria by carrying virulence-related genes and facilitating their horizontal gene transfer [34]. Using the PHASTER online tool, four prophages were identified in strain D4’s genome. The number of prophages in nine ST251 strains and the environmental isolate ATCC 7966^T^ ranged from two to five. As shown in Figure 5B, eleven distinct prophage types were detected across all strains. Notably, prophages present in ST251 strains were absent in ATCC 7966^T^. The prophage profiles were consistent among the American strains, while significant variations were observed in the Chinese strains.

The *Entero_Mu* prophage was conserved in all ST251 strains and encoded a regulatory protein (AhyD4_15485) homologous to PtrR from *Pseudomonas aeruginosa*, which has been linked to pathogenesis and antimicrobial resistance [35]. This suggests that PtrR-like proteins in ST251 may contribute to virulence, though experimental validation is needed. Additionally, the *Entero_Mu* prophage harbored genes encoding the M and S subunits of a type I restriction–modification system and secretion activator proteins (*AhyD4_15495* and *AhyD4_15500*, respectively, which are putative virulence determinants in *A. hydrophila* [36]. The conservation of *Entero_Mu* in ST251 strains and its association with virulence-related genes suggest a role in horizontal gene transfer-mediated virulence evolution.

#### 3.3.4. Genomic Island Analysis

Genomic islands (GIs) are segments of genomic DNA acquired through horizontal gene transfer and play a crucial role in bacterial environmental adaptation [37]. In this study, IslandViewer 4 was used to predict GIs in the genomes of ten *A. hydrophila* strains.

The number of GIs in the epidemic strains was significantly higher than that in the environmental strain ATCC 7966^T^. Moreover, most of the GIs present in the epidemic strains were absent in ATCC 7966^T^. All nine epidemic strains contain 14 GIs, among which GI 11, 18, 21, and 22 are functional GIs (Figure 3A).

**Figure 6:**
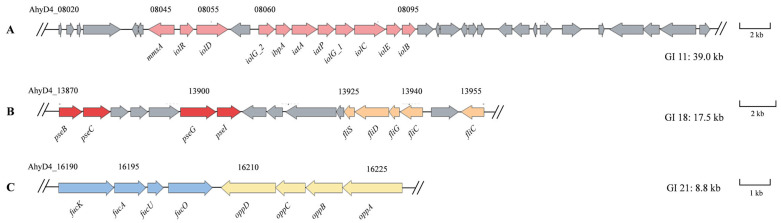
Some gene clusters in GI 11, GI 18, and GI 21. (**A**) The pink arrow indicates the *myo*-inositol utilization cluster harbored in the GI 11 region. (**B**) The red and orange colors indicate the pseudaminic acid biosynthesis and flagellar assembly gene cluster harbored in the GI 18 region. (**C**) the blue and yellow colors indicate the L-fucose metabolic pathway and a peptide ABC transport system harbored in the GI 21 region. The gray arrow indicates the genes surrounding the gene cluster.

The GI 11 region harbors a *myo*-inositol utilization cluster (Figure 6A). *Myo*-inositol can serve as a carbon source to enhance the colonization ability of bacteria in the host, thus affecting the virulence of bacteria [38]. This cluster encodes three ribose transport proteins (RbsABC) responsible for *myo*-inositol transport and eight proteins (IolARDG_2G_1CEB) involved in *myo*-inositol catabolism (Figure 6A) [39,40]. The *myo*-inositol utilization cluster in GI 11 encodes all the enzymes necessary for *myo*-inositol utilization, except 2-deoxy-5-keto-D-gluconic acid 6-phosphate aldolase, which is required for the degradation of *myo*-inositol to acetyl-CoA. Nevertheless, the D4 genome can encode homologs of 2-deoxy-5-keto-D-gluconic acid 6-phosphate aldolase, such as tagatose-bisphosphate aldolase (AhyD4_19035) and class II fructose-bisphosphate aldolase (AhyD4_19150).

GI 18 encodes all the enzymes required for pseudaminic acid biosynthesis, except PseH (Figure 6B) [41]. Pseudaminic acid can enhance the adhesion and motility of bacteria by participating in the modification of the bacterial surface and the glycosylation of flagellin proteins, thereby improving their colonization, survival, and immune evasion efficiency within the host [42]. A search of the D4 genome identified some genes predicted to encode homologs of PseH, such as GNAT family N-acetyltransferase (AhyD4_20800). Additionally, GI 18 encodes five proteins (FliS, FliD, and three flagellins) associated with flagellar assembly.

GI 21 encodes eight proteins involved in part of the L-fucose metabolic pathway and a peptide ABC transport system (OppABCD) (Figure 6C). Pathogens can utilize L-fucose as a carbon source and make use of its metabolic intermediates to supply energy or synthesize virulence factors such as the O antigen in lipopolysaccharide (LPS) [43,44]. The complete L-fucose metabolic pathway involves eight proteins encoded by the *fucRPIKAUO* operon. Typically, a regulator (FucR), a permease (FucP), a fucose mutarotase (FucU), an isomerase (FucI), a kinase (FucK), an aldolase (FucA), and a reductase (FucO) are required to produce 1,2-propanediol and dihydroxyacetone phosphate, which enter the glycolytic pathway [17]. GI 21 encodes FucK, FucA, FucU, and FucO, while the three genes *fucR*, *fucP*, and *fucI* were found upstream of GI 21.

GI 22, the largest among the nine epidemic-associated unique GIs with 41 ORFs, comprises hypothetical proteins, a DNA helicase, a DNA repair protein, a transcriptional regulator, an iron complex transport system, and a transposase.

#### 3.3.5. Two-Component Regulatory System Profiling

Two-component regulatory systems (TCSs) are conserved bacterial signaling networks critical for environmental adaptation [45]. As shown in Table 3, the TCSs were highly conserved among ST251 strains, including D4. Notable exceptions were observed in the environmental isolate ATCC 7966^T^, which lacked the phosphoglycerate transport regulator PgtC and the vancomycin resistance sensor VanS. Conversely, the magnesium-citrate transport regulator CitT was uniquely present in ATCC 7966^T^.

KEGG annotation identified 61 TCSs in ST251 strains, including virulence-associated systems ArlS/ArlR and Ihk/Irr [46]. The conservation of ArlS/ArlR and Ihk/Irr in ST251 may underlie their hypervirulence by modulating biofilm formation and stress responses [47,48].

#### 3.3.6. Plasmid Content and Functional Analysis

Plasmids are self-replicating genetic elements that confer accessory traits to microorganisms, including toxin production, stress tolerance, and anabolic pathways [49]. As shown in Table 1, among nine ST251 strains and the environmental isolate ATCC 7966^T^, only D4 and JBN2301 harbored plasmids (four and three plasmids, respectively). The genomic features of these seven plasmids are detailed in Table 4.

**Figure 7:**
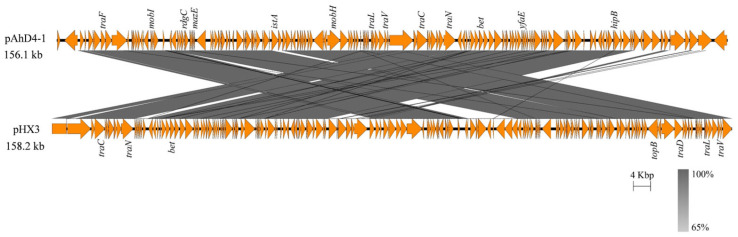
Collinear comparison of the plasmid maps between pAhD4-1 and pHX3.

Notably, three small plasmids in D4 (pAhD4-2, pAhD4-3, and pAhD4-4) shared 99–100% similarity with those in JBN2301 (pAhJBN2301-1, pAhJBN2301-2, and pAhJBN2301-3). The largest plasmid, pAhD4-1, displayed 81% similarity to pHX3 from *A. veronii* strain HX3. pAhD4-1 encoded 164 genes, including putative virulence and resistance determinants such as the fluoroquinolone resistance protein, the antitoxin MazE, the antitoxin HipB, and conjugal transfer pilus assembly proteins (Figure 7; homologous to type IV secretion system components) [8]. The presence of type IV secretion system homologs in pAhD4-1 suggests potential roles in interbacterial competition or host colonization. The three small plasmids contained 6–11 CDSs, with most annotated as hypothetical proteins.

## 4. Discussion

In recent years, MAS has emerged as a significant threat to global aquaculture, causing substantial economic losses in China, the United States, and other regions [4]. *A. hydrophila* ST251 has been identified as a hypervirulent clonal group associated with MAS outbreaks in both China and the Southeastern United States [5]. Here, we characterized the virulence phenotype and complete genome of *A. hydrophila* D4, which was isolated from diseased *M. amblycephala* and belonging to ST251, and performed comparative genomic analyses with nine ST251 strains and the environmental isolate ATCC 7966^T^ (ST1).

### 4.1. Phenotypic and Genomic Correlates of Virulence

Phenotypic characterization revealed obvious differences between D4 and ATCC 7966^T^. D4 exhibited stronger swarming motility, which aligns with it having intact flagellar assembly genes (e.g., *fliS*, *fliD*, and three flagellin genes in GI 18) critical for motility [42]. Strain ATCC 7966^T^ lacks these genes, consistent with its reduced motility.

Additionally, D4 showed significantly higher hemolytic activity and protease production, likely stemming from plasmid-encoded type IV secretion system homologs (e.g., conjugal transfer pilus proteins in pAhD4-1) that facilitate the secretion of virulence factors [8].

### 4.2. Biofilm Formation and Functional Redundancy

Contrary to expectations, both D4 and ATCC 7966^T^ formed robust biofilms with no statistically significant difference (*p* > 0.05; Figure 1E). This unexpected finding suggests that despite genomic differences, both strains employ distinct strategies to achieve comparable biofilm capacities. D4’s biofilm formation capabilities may rely on ST251-specific ABC transporters and the L-fucose metabolic pathway, which facilitate adhesion to host tissues [50]. Additionally, the PtrR-like regulator encoded by the prophage *Entero_Mu* in D4 may upregulate biofilm-associated genes, as observed in *P. aeruginosa* [35]. In contrast, ATCC 7966^T^ lacks these pathways but harbors CitT, a magnesium citrate transport regulator unique to ATCC 7966^T^. This suggests that ATCC 7966^T^ may utilize alternative carbon sources (e.g., citrate) to form biofilms, aligning with its adaptation to aquatic or other environments. The comparable biofilm capacities of D4 and ATCC 7966^T^ highlight functional redundancy in biofilm formation mechanisms. While D4 relies on host-derived nutrients (e.g., L-fucose), ATCC 7966^T^ may utilize environmental resources (e.g., citrate), indicating their distinct ecological niches. This divergence in metabolic strategies likely contributes to their differential virulence phenotypes despite similar biofilm formation abilities.

### 4.3. Genomic Insights into ST251 Pathogenesis

Comparative genomic analysis revealed that the environmental isolate ATCC 7966^T^ harbored extensive CDS deletions compared to ST251 strains, suggesting reduced functional complexity (Figure 3A). The ANI among nine ST251 *A. hydrophila* strains ranged from 99.74% to 100%, which is significantly higher than the values observed in other *A. hydrophila* lineages (96.24–96.97%) (Figure 2). Phylogenetic analysis confirmed close genetic relationships within the ST251 clade, with strains from the same geographic region forming even tighter clusters. Genome collinearity analysis further supported this finding (Figure 3B).

*A. hydrophila* is known to harbor diverse virulence determinants, including secretion systems and flagellar apparatus, which are often recognized as critical virulence determinants [7]. Notably, VFDB annotation revealed no significant differences in core virulence gene repertoires among ten *A. hydrophila* genomes, except for T6SS. Strikingly, T3SS and lateral flagella were absent in all ten strains, including ST251 isolates (Figure 5A). This suggests that these systems are non-essential for ST251 pathogenesis.

To identify unique virulence determinants in ST251, COG and KEGG functional annotations were performed (Figure 4A). The analysis revealed that ST251 genomes are enriched in genes encoding transposases, integrases, ABC transporters, and enzymes involved in the *myo*-inositol and L-fucose metabolic pathways (Figure 4B). These findings imply that ST251 may rely on metabolic adaptation rather than classical virulence systems to establish infection.

### 4.4. Mobile Genetic Elements and Adaptive Evolution

Plasmids and prophages in ST251 strains may contribute to their virulence, resistance, and adaptation to the environment [51,52]. D4’s plasmid pAhD4-1 encodes type IV secretion system homologs and fluoroquinolone resistance determinants, suggesting roles in horizontal gene transfer and antimicrobial resistance. The conserved prophage *Entero_Mu* in ST251 (Figure 5B), which is absent in ATCC 7966^T^, encodes a PtrR-like regulator (AhyD4_15485) linked to *P. aeruginosa* pathogenesis [35]. This protein may enhance ST251’s ability to colonize host tissues by modulating stress responses.

GI analysis identified 26 GIs (4–54 kb) in *A. hydrophila* D4 (Figure 3A). Fourteen GIs were considered as ST251-specific virulence-associated islands, and they were absent in the environmental isolate ATCC 7966^T^ but conserved in ST251 strains. Notably, two of these GIs harbored genes encoding the *myo*-inositol and L-fucose metabolic pathways, while another contained a pseudaminic acid biosynthesis cluster.

### 4.5. Metabolic Adaptation as a Pathogenic Strategy

Bacterial metabolic adaptability is critical for survival and proliferation in diverse environments. Studies in pathogenic *Salmonella*, *Vibrio*, and *Helicobacter* have demonstrated that metabolic genes are as essential as classical virulence factors during infection [53]. ST251 strains encode two distinct metabolic pathways (*myo*-inositol and L-fucose) and a pseudaminic acid biosynthesis cluster, which may collectively contribute to their hypervirulence.

*Myo*-inositol, abundant in the fish liver and intestine, may serve as a carbon source during infection [29]. D4 encodes a complete *myo*-inositol utilization cluster (GI 11), including *iolR*, which regulates aerolysin expression [40]. This suggests a nutrient-dependent regulatory axis where *myo*-inositol availability could enhance virulence factor production, conferring a competitive advantage in host tissues.

L-fucose, a terminal residue of intestinal mucins, may serve as both an adhesion target and an energy source [54,55]. Fucosylated glycans facilitate bacterial colonization by interacting with host lectins [56], potentially promoting ST251 persistence in mucosal environments.

Notably, ST251 strains uniquely encode pseudaminic acid biosynthesis genes (GI 18). This non-canonical sugar modifies flagellin, enabling functional flagellar assembly and motility [42]. Since pseudaminic acid is restricted to pathogenic bacteria, this pathway likely contributes to ST251’s enhanced motility and host colonization capacity.

Collectively, these metabolic pathways and biosynthesis systems represent novel virulence determinants in ST251. By exploiting host-derived nutrients (*myo*-inositol and L-fucose) and optimizing motility via pseudaminic acid, ST251 may outcompete environmental isolates and establish infection. This metabolic plasticity, which is absent in ATCC 7966^T^, likely underlies the hypervirulence of the ST251 clonal group.

### 4.6. Limitations and Future Research Directions

This study provides initial insights into the pathogenicity and environmental adaptability of the ST251 epidemic strain; however, further investigations are needed to validate and expand upon the current findings:

(1) Constructing deletion or overexpression mutants of key virulence or regulatory genes (e.g., pse genes, *PtrR*, *IolR*), combined with in vitro and in vivo infection models, will help clarify their roles in motility, biofilm formation, and virulence expression.
(2) Transcriptomic analyses under various environmental stress conditions (e.g., high temperature and salinity and nutrient limitation) and natural host infection models (e.g., blunt snout bream) could provide mechanistic insights into transcriptional regulation and virulence adaptation in complex environments. Integrating histopathology and host immune gene expression will further improve ecological relevance.
(3) Expanding the strain collection to include more ST251 isolates from diverse geographic regions and host sources, along with host response analyses (e.g., host transcriptomics, inflammatory markers, and immune cell activation), will facilitate a systems-level understanding of host–pathogen interactions and their impact on infection outcomes.

Future research integrating genome editing, multi-omics approaches, and ecologically relevant infection models will be crucial for elucidating the molecular mechanisms underlying ST251 virulence evolution and environmental adaptation, ultimately supporting the development of effective control strategies for aquaculture-associated outbreaks.

## 5. Conclusions

This study provides valuable insights into the genomic and phenotypic features of *A. hydrophila* ST251, a hypervirulent lineage responsible for widespread outbreaks of MAS in aquaculture. Phenotypic analyses revealed that, compared to the environmental isolate ATCC 7966^T^, strain D4 exhibited significantly enhanced motility, hemolytic activity, and protease activity, as well as increased virulence in the zebrafish infection model, suggesting greater pathogenic potential.

Comparative genomic analysis of D4, nine additional ST251 strains, and ATCC 7966^T^ showed that ST251 strains form a distinct phylogenetic clade with an ANI score above 99.74%. Their genome sizes and the number of CDSs were generally larger than those of ATCC 7966^T^. Although the G + C content remained consistent, extensive genome rearrangements were observed among ST251 strains from China and the United States, as well as between ST251 and environmental strains.

Notably, ST251-specific genomic islands encode pathways for inositol and L-fucose utilization and a pseudaminic acid biosynthesis gene cluster, which may enhance metabolic versatility and host colonization by enabling nutrient acquisition and flagellar glycosylation. In addition, although typical virulence factors such as T3SS, T6SS, RTX clusters, and lateral flagella are commonly present in ST251 strains, their absence or variation in some isolates suggests that these factors may not be the core determinants of the heightened virulence of this lineage. Moreover, the types of secondary metabolite gene clusters showed no significant differences between ST251 and environmental strains, implying that these clusters are unlikely to be major drivers of virulence enhancement.

Prophage analysis revealed that ST251 strains universally harbor a conserved *Entero_Mu* prophage encoding a PtrR-like transcriptional regulator that is homologous to proteins involved in antimicrobial resistance and virulence in *P. aeruginosa*. Additionally, conserved two-component regulatory systems such as ArlS/ArlR and Ihk/Irr were identified, which may contribute to increased environmental adaptability and pathogenicity by enhancing stress responses and biofilm formation. The detection of type IV secretion system homologs on plasmid pAhD4-1 further suggests possible roles in interbacterial competition and host colonization.

In summary, these findings provide novel insights into the molecular mechanisms underlying the pathogenicity and environmental adaptability of hypervirulent *A. hydrophila* ST251 strains.

## Figures and Tables

**Table 1:** General features of *A. hydrophila* genomes.

	D4	JBN2301	ZYAH72	NJ-35	J-1	GYK1	ML09-119	AL09-71	pc104A	ATCC 7966^T^
Accession No.	CP013965	CP013178	CP016989	CP006870	CP006883	CP016392	CP005966	CP007566	CP007576	CP000462
Date of isolation	2012	2009	2015	2010	1989	2001	2009	2009	2010	1901
Location	Wuhan, China	Wuhan, China	Wuhan, China	Nanjing, China	Nanjing, China	Guangzhou, China	Mississippi State USA	West Alabama USA	West Alabama USA	USA
Host/source	Diseased Fish (*Megalobrama amblycephala*)	Diseased Fish (*Carassius auratus*)	Diseased Fish *(Carassius auratus*)	Diseased Fish (*Carassius auratus*)	Diseased Fish (*Carassius auratus*)	Diseased Fish (*Siniperca chuatsi*)	Diseased Fish (*Ictalurus punctatus*)	Diseased Fish (*Ictalurus punctatus)*	Environment (Soil of a Catfish Pond)	Food (Fishy milk)
Genome size (bp)	5,100,520	5,127,362	5,159,182	5,279,644	5,000,814	4,951,765	5,024,500	5,023,861	5,023,829	4,744,448
G + C Content (%)	60.80	60.78	60.70	60.50	60.90	60.80	60.80	60.80	60.80	61.51
CDS	4569	4438	4397	4526	4268	4219	4446	4297	4300	4151
rRNAs	31	31	31	31	31	31	31	31	31	31
tRNAs	117	129	123	102	110	114	112	111	111	126
ncRNAs	7	1	7	2	2	8	7	2	2	5
Pseudo Genes	50	47	59	55	51	43	102	51	49	31
Plasmid	4	3	-	-	-	-	-	-	-	-

**Table 2:** Gene clusters of secondary metabolites of ten *A. hydrophila* strains.

Cluster ID	Cluster Type	Most Similar Known Cluster	Similarity (%)	D4	ZYAH72	NJ-35	J1	GYK1	JBN2301	ML09-119	AL09-71	Pc104A	ATCC 7966^T^
Cluster1	Arylpolyene	Aryl polyenes, other	61%										
Cluster2	Bacteriocin												
Cluster3	NRPS	Amonabactin P 750, nrps	100%										
Cluster4	Hserlactone												
Cluster5	Bacteriocin												

The blue cells indicate that the strain harbors the corresponding gene cluster, while the gray cells indicate its absence.

**Table 3:** Comparison of TCSs from ten *A. hydrophila* strains.

Strains	TCS Family/Number									
	OmpR Family	NarL Family	NtrC Family	Chemotaxis Family	Cellcycle Family	LuxR Family	Lux Family	LytTR Family	CitB Family	Sporulation Family
D4	69	22	22	22	7	6	4	6	9	5
ZYAH72	69	22	22	22	7	6	4	6	9	5
NJ-35	69	22	22	22	7	6	4	6	9	5
J-1	69	22	22	22	7	6	4	6	9	5
GYK1	69	22	22	22	7	6	4	6	9	5
JNB2301	69	22	22	22	7	6	4	6	9	5
ML09-119	69	22	22	22	7	6	4	6	9	5
AL09-71	69	22	22	22	7	6	4	6	9	5
pc104A	69	22	22	22	7	6	4	6	9	5
ATCC 7966^T^	68	22	21	22	7	6	4	6	10	5

**Table 4:** General feature of *A. hydrophila* D4 and JBN2301.

Plasmid	Plasmid Size (bp)	GC Content %	Numbers of CDSs	Most Similar Plasmid	Similarity %
pAhD4-1	156,086	50.12	164	pHX3	81%
pAhD4-2	6318	56.25%	11	pAhJBN2301-1	100%
pAhD4-3	6163	54.28	6	pAhJBN2301-2	99%
pAhD4-4	6045	51.50%	9	pAhJNB2301-3	100%
pAhJBN2301-1	6318	56.25%	11	pAhD4-2	100%
pAhJBN2301-2	6162	54.28%	6	pAhD4-3	99%
pAhJBN2301-3	6045	51.50%	9	pAhD4-4	100%

## Data Availability

The original contributions presented in this study are included in the article. Further inquiries can be directed to the corresponding authors.

## Abbreviations

The following abbreviations are used in this manuscript:

ANI	Average nucleotide identity
*A. hydrophila*	*Aeromonas hydrophila*
CAS	Chrome azurol-S
CDSs	Coding sequences
MAS	Motile *Aeromonas* Septicemia
PBS	Phosphate-buffered saline
T3SS	Type III secretion system
T6SS	Type VI secretion system
ST251	Sequence type 251
